# Medical Treatment of Hyperthyroidism; Efficacy and Safety Considerations

**DOI:** 10.34172/aim.33502

**Published:** 2025-12-01

**Authors:** Fereidoun Azizi, Hengameh Abdi, Seyed Alireza Ebadi, Ladan Mehran, Atieh Amouzegar

**Affiliations:** ^1^Endocrine Research Center, Research Institute for Endocrine Sciences, Shahid Beheshti University of Medical Sciences, Tehran, Iran; ^2^Department of Internal Medicine, School of Medicine, Shahid Beheshti University of Medical Sciences, Tehran, Iran

**Keywords:** Antithyroid drug, Efficacy, Hyperthyroidism, Safety

## Abstract

Antithyroid drugs (ATDs) are often the first treatment option for hyperthyroidism due to their efficacy and safety profile. Long-term ATD treatment can effectively control hyperthyroidism and prevent relapse. In this review, we summarize the findings of clinical trials and clinical experiences on the use of ATD treatment for hyperthyroidism. We discuss the efficacy and safety of ATD treatment, as well as the optimal duration of treatment. The evidence suggests that ATD therapy is selected as initial therapy, treatment of relapse of hyperthyroidism and in patients with persistent elevation of TSH receptor antibodies after 18 months of ATD therapy. Long-term ATD treatment can be an effective and safe option for management of many patients with hyperthyroidism. However, additional studies are needed to establish the most efficacious treatment duration and to identify patients who are most likely to benefit from long-term ATD treatment.

## Introduction

 Hyperthyroidism, a widespread endocrine disease, is defined by the excessive synthesis and release of thyroid hormones from an overactive thyroid gland. Graves’ disease is the most common cause of hyperthyroidism. It is an autoimmune disorder characterized by the production of thyroid-stimulating immunoglobulins. Hyperthyroidism due to toxic adenoma and toxic multinodular goiter is less common. Other less frequent causes of hyperthyroidism include trophoblastic disease, thyrotropin-secreting pituitary adenoma, and resistance to thyroid hormone. In addition, there are rare causes of thyrotoxicosis without hyperactivity of thyroid gland, in which the radioactive iodine uptake of thyroid is low; these include several types of thyroiditis, iatrogenic and factitious thyrotoxicosis, struma ovarii and metastasis from follicular thyroid cancer.^1,2^

 Common modalities for treatment of hyperthyroidism include medical treatment with thionamide compounds (antithyroid therapy), radioactive iodine administration and thyroidectomy. High doses of radioiodine and total thyroidectomy have been recommended for treatment of hyperthyroidism causing lifelong hypothyroidism.^1^ Antithyroid drugs (ATDs), which inhibit the production of thyroid hormones, are the first-line therapeutic modality for the majority of patients with hyperthyroidism. The two most commonly used ATDs are methimazole and propylthiouracil. Short-term ATD therapy is effective in controlling hyperthyroidism,^3^ but long-term therapy may be necessary to achieve remission.^4^

 The optimal duration of ATD treatment for Graves’ hyperthyroidism remains unclear. Some studies have indicated that long-term therapy may be necessary to achieve remission, while others have shown that short-term therapy is sufficient.^5-8^ The American Thyroid Association (ATA) recommends that ATD therapy should be continued for at least 12-18 months, but the optimal duration of treatment beyond this period is uncertain.^1^ Although some studies had shown that ATD treatment up to 4 years had no benefit for increasing the remission rate,^9^ recent studies document higher remission rates with long-term ATD treatment of ≥ 5 years.^10^

 ATD adverse events, such as agranulocytosis and hepatotoxicity, are rare.^11^ Agranulocytosis is a rare but serious side effect that can lead to life-threatening infections, while hepatotoxicity can cause liver damage and dysfunction. The risk of these adverse events markedly falls following the first year of continuous long-term ATD therapy.^12^

 This review aimed to summarize the findings of clinical trials and personal clinical experiences regarding the use of medical treatment for hyperthyroidism. We will discuss the efficacy and safety as well as the optimal duration of treatment. We will also compare medical treatment with other treatment options and identify patients who are most likely to benefit from ATD treatment.

## Search Strategy

 We conducted a literature search using PubMed from January 1, 1980 to August 30, 2024 to identify clinical trials and personal clinical experience reports on medical treatment for Graves’ hyperthyroidism. The search terms used included “Graves’ hyperthyroidism”, “nodular (multinodular) toxic goiter”, “toxic adenoma”, “radioactive iodine”, “thyroidectomy”, “antithyroid drugs”, “long-term treatment”, and “clinical trials”. We included studies that reported on the efficacy and safety of various treatment modalities for hyperthyroidism, as well as personal clinical reports that provided insight into the management of hyperthyroidism.

 We included clinical trials and observational studies which (1) evaluated the effects of various modalities of therapy on hyperthyroidism, (2) had a follow-up period of at least 12 months, and (3) reported outcomes such as remission rates, relapse rates, adverse events, or quality of life (QOL). We also included the results of the largest cohort of long-term ATD treatment in 1163 patients, named Towards Outstanding Hyperthyroid Care Induced by Antithyroid Drugs (TOHID).^13,14^

 Treatment of thyrotoxicosis should be directed at its cause. Hyperthyroidism due to overactivity of the thyroid gland is commonly caused by Graves’ disease, toxic multinodular goiter and toxic adenoma; the fundamental causes of these conditions are unknown. Therefore, the main aim of treatment is directed at inhibiting thyroidal hormone synthesis and release or destroying thyroid tissue by radioiodine or thyroidectomy.^11^

 Thionamide compounds have been used as effective antithyroid medications in the last 80 years for Graves’ hyperthyroidism and preparation of many patients with nodular toxic disease before ablation. ATDs have been chosen as the first-line therapeutic modality of hyperthyroidism in the last two decades and they have become a mainstay of treatment of patients with hyperthyroidism.^15,16^

 Patients who have contraindication for ATD use or fail to become euthyroid during ATD therapy, those with comorbidities that increase the surgical risk, and patients with recurrent thyrotoxicosis after prior thyroidectomy may be considered as good candidates for radioiodine therapy. Women who prefer thyroidectomy before planning for pregnancy, patients with symptomatic compression or mega goiters, those suspected for thyroid malignancy, coexisting hyperparathyroidism and selected patients with severe active Graves’ orbitopathy should also be considered for thyroidectomy.^1,17,18^

## Appropriate Duration of ATD Treatment

 Since the introduction of ATD for treatment of hyperthyroidism,^19^ the appropriate duration of therapy has been a matter of debate. The duration of treatment was variable between 6-24 months in the 1950s through 1980s.^20^ Abraham et al, based on two studies (12 vs. 24 months and 18 vs. 42 months of ATD treatment) concluded that ATD treatment for more than 18 months had no added benefit regarding remission rate and proposed that 12-18 months of treatment was optimal.^21^ However, approximately 50% (range 20%-70%) of patients would experience recurrence of hyperthyroidism following cessation of 18-20 months of treatment with ATD.^22^

## Efficacy of Long-term ATD Treatment for Hyperthyroidism

 Several clinical trials have revealed that long-term ATD treatment can effectively control hyperthyroidism and prevent relapse.^23-29^ For example, a clinical trial conducted by Azizi et al found that long-term methimazole was effective in controlling hyperthyroidism and preventing relapse in patients with Graves’ disease.^10^ The study followed patients for up to 4 years after discontinuation of methimazole treatment and reported significant decrease relapse rate in patients who had 60 months of ATD therapy as compared to those with 18.8 months of ATD treatment (15% vs 53%, *P* < 0.001).

 Two systematic review/meta-analyses have shown the effectiveness and safety of long-term ATD therapy.^30,31^ Long-term therapy has been defined as constant ATD therapy for ≥ 60 months, since shorter duration up to 48 months of therapy could not increase the remission rate higher than that observed by 12-18 months of ATD treatment.^8,9^ Treatment with ATD for > 60 months has been shown to be effective in controlling hyperthyroidism and preventing relapse.^4^

## Safety of Long-term ATD Therapy

 Adverse events of ATD therapy consist of minor allergic side effects such as pruritus and allergic reactions and rare but major adverse events of agranulocytosis, hepatitis and vasculitis.

 Agranulocytosis is a rare but potentially life-threatening condition characterized by a low blood cell count. The risk of agranulocytosis is highest in the first few months of treatment and decreases with longer duration of treatment.^12^ Regular monitoring of blood cell counts is recommended during ATD treatment.^1^ Long-term methimazole treatment is generally considered safe, with few serious adverse effects reported. A systematic review including data from 1660 patients with a mean duration of ATD therapy of 5.8 years reported the rate of minor complications at 2-36% and the occurrence of major adverse events in only 14 patients (7, 5, 1 and 1 cases of agranulocytosis, liver damage, glomerulonephritis and vasculitis, respectively).^31^ All but one major adverse events occurred in the first year of treatment. The only major complication after the first year was a case of vasculitis due to propylthiouracil (Table 1). In a prospective multicenter study in Denmark, Karmisholt et al reported that in 208 patients with Graves’ hyperthyroidism, 10% of patients experienced adverse events and 75% of the cases occurred during the first months of ATD therapy. After 24 months, the dose of methimazole was reduced to 5 mg daily and no further adverse event occurred up to 48 months of treatment.^32^

**Table 1:** Minor and Major Adverse Events in 1660 Patients Treated with Antithyroid Drugs for a Mean of 5.8 Years

**Adverse effects**	**Duration of ATD treatment**	
	**Up to one year**	**After 12 months**
Minor	123	4
Cutaneous	74	
Elevated liver enzymes	9	
Arthralgia	5	
Myalgia	2	
Thrombocytopenia	2	
Fever	2	
Nausea	2	
Oral aphthous	1	
Major	14	1^*^

^*^ANCA-associated glomerulonephritis due to propylthiouracil treatment.

## Optimal Duration of Treatment

 The optimal duration of ATD treatment for Graves’ hyperthyroidism is still a matter of debate. Some studies have suggested that long-term treatment (i.e. more than 48 months) may be necessary to achieve remission and prevent relapse.^30,33-35^ The optimal treatment duration depends on several factors, including the baseline GREAT score based on the patient’s age, serum concentrations of free thyroxine and TSH receptor antibodies (TRAb) and goiter size as well as serum TRAb at 18 months.^36^

**Figure 1:**
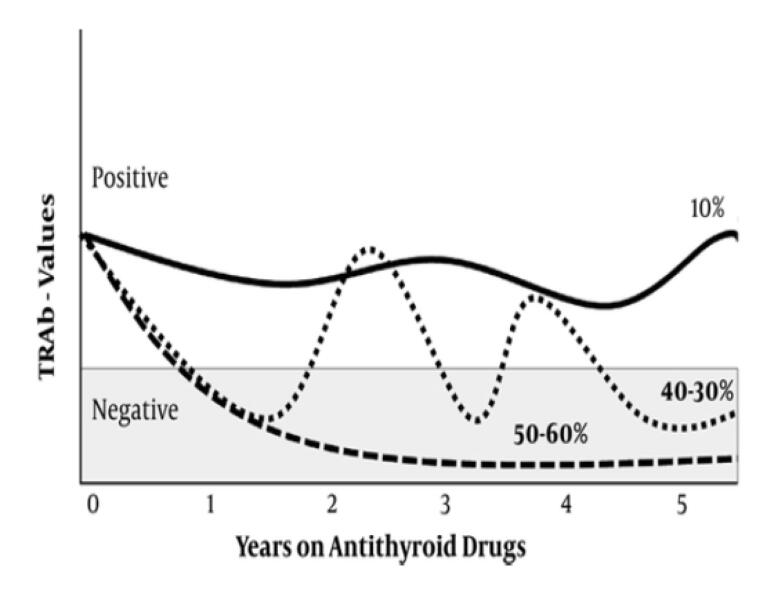
Trends in Serum TSH Receptor Antibodies (TRAb) and Remission Rate During Years of Antithyroid Drug Treatment. Curves demonstrate trends of TRAb values during ATD treatment. In 50%-60% of patients, TRAb decreases to normal values after 1-2 years of therapy and is associated with high rates of remission. Persistently high TRAb concentration in 10% and fluctuating TRAb concentrations in 30%-40% are probably responsible for the high relapse rate in approximately 50% of patients. Increase in remission rate occurs with ATD therapy for more than 5 years, which is accompanied by normalization of TRAb in the majority of fluctuating group. Reprinted from Villagelin D, Santos RB, Romaldini JH. Remission rate of Graves’ disease and the trend of changes in serum TSH receptor antibodies in prolonged antithyroid drug treatment. Int J Endocrinol Metab. 2020;18(Suppl):e101473. https://doi.org/10.5812/ijem.101473. (The Creative Commons Attribution-NonCommercial 4.0 International License (http://creativecommons.org/licenses/by-nc/4.0/).

 It has been shown that during ATD therapy, high serum TRAb may persist in 10% and fluctuate in another 30%-40%^35^ (Figure 1). Therefore, remission may not occur in half of patients with Graves’ disease before five years of ATD treatment. The fall of TRAb levels to low normal values or disappearance of TRAb in the following years increases the chance of disease remission. Therefore, it is recommended that long-term ATD treatment should be continued until serum TSH concentration increases to normal values and TRAb levels are undetectable or in the low normal range.^14,33,37^

**Figure 2:**
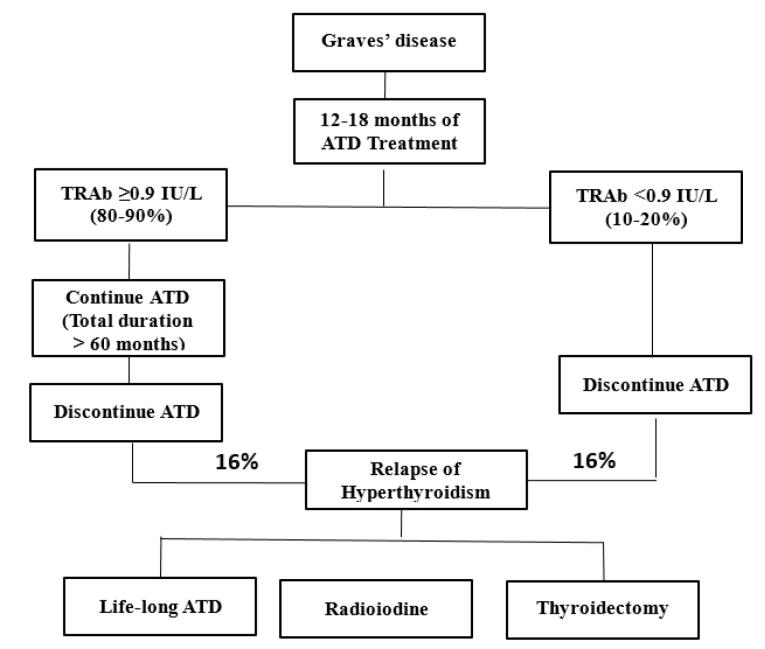
Algorithm for Management of Graves’ Disease. After 12-18 months of antithyroid drug (ATD), treatment should be discontinued in those with low serum TSH-receptor antibody (TRAb) levels; ATD treatment should be continued in 80-90% of patients who have normal or high TRAb titers ( ≥ 0.9 IU/L) for at least 60 months. Following treatment withdrawal, in both arms, approximately 16% of patients may have relapse of hyperthyroidism. Such patients may be treated with life-long ATD or ablation therapies. Adopted from Abraham et al^9^ and Azizi et al^10^

 Identification of patients who are most likely to benefit from long-term ATD treatment is an important clinical challenge. It is recommended that after 12 to 18 months of ATD therapy, an evaluation of clinical improvement of the patient and normalization of serum TSH and TRAb concentrations is made before deciding upon discontinuation or continuous ATD long-term treatment.^14^ ATD should be discontinued only in those patients with TRAb levels < 0.9 IU/L, at the end of 12-18 months of ATD therapy.^36^ The remaining 80%-90% of patients have normal or elevated TRAb and should be continued on long-term ATD for > 60 months (Figure 2). A practical scoring of patients under long-term ATD therapy for discontinuation of treatment has been recently published and may be considered in patients on long term-ATD therapy for discontinuation of medical therapy.^38^ This model, which incorporates variables such as age, sex, goiter grade, and serum levels of fT4, T3, TSH, and TRAb, generates a score from 0 to 14. Based on this score, patients are stratified into risk categories: low risk of relapse ( < 20%) for scores < 8 and high risk ( > 60%) for scores of 11-14.

## Comparison of Long-term Antithyroid Treatment with other Treatment Options

 Long-term methimazole treatment is one of several treatment options available for Graves’ hyperthyroidism. Radioiodine therapy and surgery are also effective treatment options, but they are associated with a higher risk of hypothyroidism and other complications.^1^

 Randomized clinical trials in Iran have shown that in patients with recurrent Graves’ disease, long-term methimazole therapy is superior to radioactive iodine ablation (Table 2), because of lower cost, better lipid profile, lower risk of abnormal TSH values and better echocardiographic parameters.^23,39^ It is noteworthy that 61-69% of patients may experience recurrent hyperthyroidism after sub-ablative doses of radioiodine^40^ and ablative doses cause lifelong hypothyroidism requiring levothyroxine consumption, which may be associated with impaired resting energy expenditure, QOL and psychological well-being.^41-44^

**Table 2:** Comparison of Effectiveness of Long-term Antithyroid Drug (LT-ATD) Versus Radioactive Iodine Treatment (RAI) for Graves’ Hyperthyroidism

**Outcome**	**LT-ATD vs RAI**
Shorter time to biochemical improvement	Better
Sustained euthyroidism	Better
Echocardiographic indices of velocity	Better
Quality of life	Better
Recurrence of hyperthyroidism	Less
Abnormal TSH at various times	Less
Worsening of thyroid eye disease	Less
Increased body mass index	Less
Lipid profile derangement	Less
Overall cost	Less

 Six years after subtotal thyroidectomy for hyperthyroidism, 29% of patients had persistent or recurrent thyrotoxicosis and 21% and 50% were euthyroid and hypothyroid, respectively^45^; therefore, the ATA and European Thyroid Association recommend total thyroidectomy, which results in permanent treatment for hyperthyroidism in the majority of patients.^2,46^

## Choice of Treatment Considering Cardiovascular Outcomes and Mortality

 Increased all-cause mortality is seen in patients with hyperthyroidism.^47^ Uncontrolled hyperthyroidism may accompany increased risk of mortality and cardiovascular morbidity,^48^ and longer duration of suppressed serum TSH levels increases cardiovascular outcomes in both untreated and treated patients.^49,50^ Therefore, cardiovascular safety must be considered during the management of hyperthyroidism, since early and effective control of hyperthyroidism improves overall survival.^50^

 It has been shown that time to euthyroidism and time remained in euthyroidism favor long-term ATD therapy over radioactive iodine treatment.^51^ Steady normalization of serum TSH during ATD therapy may be important for prevention of cardiovascular complication in patients with hyperthyroidism.^52^

## Discussion

 ATDs are the first-line treatment for hyperthyroidism, but the optimal duration of therapy remains unclear. While short-term ATD therapy is effective in controlling hyperthyroidism, long-term therapy may be necessary to achieve remission.^2,4^

 The results of this review suggest that long-term ATD therapy is associated with higher remission rates than the short-term regimen and demonstrates a favorable safety and efficacy profile. This finding is consistent with previous reports that have shown that long-term therapy is necessary to achieve remission in some patients with hyperthyroidism.^30,34^ The ATA recommends that ATD therapy should be continued for at least 12-18 months, but the optimal treatment duration beyond this period needs further consideration.^1^

 The higher remission rate with long-term therapy may be due to the gradual reduction in thyroid hormone production and the suppression of thyroid gland activity. Long-term therapy may also allow for the resolution of underlying autoimmune processes that contribute to hyperthyroidism. However, the optimal duration of therapy may vary depending on the patients’ clinical status, risk of adverse events, and preferences.^33,37^

 Long-term ATD therapy is associated with low risk of adverse events.^12,31^ Agranulocytosis is a rare but serious side effect that can lead to life-threatening infections, and hepatotoxicity can cause liver damage and dysfunction. The risk of these adverse events decreases with the duration of ATD therapy beyond 12 months and is seldom seen in patients on long-term therapy.^31^

 QOL was not consistently reported across studies.^23^ However, QOL is an important outcome for patients with hyperthyroidism; some studies have shown decreased QOL in hyperthyroid patients treated with radioiodine as compared to those on ATD therapy or thyroidectomy^44^; further studies are needed to evaluate the long-term effects of ATD therapy on QOL.

 The optimal duration of ATD therapy for hyperthyroidism should be individualized based on the patient’s clinical status and risk of adverse events. Clinicians should weigh the benefits and risks of long-term therapy when deciding on the optimal duration of treatment for their patients. Patients on long-term therapy should be closely monitored for adverse events, and treatment should be adjusted as necessary. These considerations and findings of many elegant studies in the management of hyperthyroidism in the past decades have caused paradigm shifts in the management of hyperthyroidism, shown by a 2023 international survey of clinical practice. The selection of initial therapy with radioiodine has decreased from 69% to 11.1% from 1990 to 2023. As many as 68.7% of responders stated that they would continue ATDs if TRAb positivity persists after 18 months of ATD therapy. After relapse of hyperthyroidism, resumption of ATD therapy was chosen by 60%. Therefore, long-term ATD therapy has been adopted as a treatment of choice by many thyroidologists for hyperthyroid patients.^53^

 This review has several limitations. First, the included studies had heterogeneity in terms of study design, sample size, and follow-up period. Second, the quality of the observational studies was not high, which may have influenced the overall effect size. Third, the studies did not consistently report the QOL outcome, which limits the ability to draw conclusions about the effects of ATD therapy on QOL. Finally, most clinical studies related to long-term ATD treatment are mainly from a single center in Iran, which is an iodine-sufficient area and may not be generalizable to other controls.

## Conclusion

 In conclusion, medical treatment with thionamide compounds has been considered as the first-line management of hyperthyroidism. The optimal duration of ATD therapy for hyperthyroidism should be individualized based on the patients’ clinical status and decrease in TRAb concentrations. Long-term ATD therapy is an effective and safe treatment option for hyperthyroidism. Clinicians should weigh the benefits and risks of long-term therapy when deciding on the optimal duration of treatment for their patients. Further studies are needed to focus on identifying reliable predictors of treatment response to long-term ATD treatment and determining the optimal duration of treatment and to evaluate the long-term effects of ATD therapy on QOL, cardiovascular outcomes, all-cause mortality and cost-effectiveness of long-term ATD therapy in comparison to other treatment modalities.
